# Development of an mRNA Vaccine for Tick-Borne Encephalitis: Selection of a Prototype Virus Strain

**DOI:** 10.3390/vaccines14010107

**Published:** 2026-01-21

**Authors:** Maria A. Nikiforova, Vladimir A. Gushchin, Denis A. Kleymenov, Anastasia M. Kocherzhenko, Evgeniia N. Bykonia, Elena P. Mazunina, Sofia R. Kozlova, Leonid I. Russu, Nadezhda A. Kuznetsova, Elena V. Shidlovskaya, Elizaveta V. Marchuk, Evgeny V. Usachev, Olga V. Usacheva, Dmitry V. Shcheblyakov, Irina V. Kozlova, Sergei E. Tkachev, Andrei A. Pochtovyi, Vladimir I. Zlobin, Denis Y. Logunov, Alexander L. Gintsburg

**Affiliations:** 1 Gamaleya National Research Center for Epidemiology and Microbiology, 123098 Moscow, Russia; mne10000let@yandex.ru (D.A.K.); kocherzhenko-an@mail.ru (A.M.K.); evgeniya_bikonya@mail.ru (E.N.B.); mazuninaelenka@yandex.ru (E.P.M.); nadyakuznetsova0@gmail.com (N.A.K.); lenitsa@gmail.com (E.V.S.); elizaveta.divisenko@yandex.ru (E.V.M.);; 2 Department of Medical Genetics and Postgenomic Technologies, Federal State Autonomous Educational Institution of Higher Education I.M. Sechenov First Moscow State Medical University of the Ministry of Health of the Russian Federation (Sechenov University), 119991 Moscow, Russia; 3 Department of Virology, Faculty of Biology, Lomonosov Moscow State University, 119234 Moscow, Russia; 4 Scientific Centre for Family Health and Human Reproduction Problems, Siberian Branch of the Russian Academy of Sciences, 664003 Irkutsk, Russia; diwerhoz@rambler.ru; 5 Institute of Fundamental Medicine and Biology, Kazan Federal University, 420008 Kazan, Russia

**Keywords:** tick-borne encephalitis virus, mRNA vaccine, cross-immunity, protective efficacy

## Abstract

**Background/Objectives**: While tick-borne encephalitis virus (TBEV) is genetically relatively conserved, the significant antigenic divergence between its main circulating subtypes hinders the development of broadly effective antiviral treatments and vaccines. Current inactivated TBEV vaccines offer limited cross-protection against heterologous strains, as evidenced by cases among vaccinated individuals in endemic regions. The aim of this study was to design a candidate mRNA vaccine and evaluate the breadth of protective immunity it elicits. **Methods**: Ten candidate mRNA-PrM/E-LNP vaccines were comparatively evaluated for immunogenicity and protective efficacy in BALB/c mice. Immunogenicity was assessed by measuring antigen-specific IgG titers via ELISA and neutralizing antibody titers against a panel of TBEV strains using a virus-neutralization test. Protective efficiency was determined in a lethal challenge model, where immunized mice were challenged with one of seven distinct TBEV strains. **Results**: Vaccination with all tested mRNA-PrM/E-LNP candidates conferred 100% survival in mice following a lethal challenge with each of the seven TBEV strains (100 LD_50_). The construct mRNA-PrM/E—Krasny Yar-8 demonstrated the highest immunogenicity, inducing antigen-specific antibodies with a geometric mean titer (GMT) of 1:6625, as well as the broadest virus-neutralizing activity against both homologous and heterologous TBEV strains in vitro. **Conclusions**: The mRNA platform represents a promising strategy for developing TBEV vaccines, demonstrating high immunogenicity and cross-protective efficacy against diverse viral strains.

## 1. Introduction

Tick-borne encephalitis (TBE) remains a major natural focal infectious disease, posing a significant public health threat across endemic regions of Europe and Asia from Eastern France to Japan and from Northern Russia to Kyrgyzstan [1,2]. The causative agent, tick-borne encephalitis virus (TBEV), belongs to the genus *Orthoflavivirus* within the *Flaviviridae* family and is primarily transmitted through bites of infected ticks from genera *Ixodes*, *Dermacentor* and *Haemophysalis*. TBEV infection can lead to severe neurological complications, including meningitis, encephalitis and persistent paralysis [3]. Traditionally, TBEV is classified into three main subtypes—Far Eastern (TBEV-FE), European (TBEV-Eu) and Siberian (TBEV-Sib). Additional genetic lineages, referred to as subtypes, have also been described, such as strain 178-79 (fourth subtype) and the Baikal subtype (TBEV-Baik). In the absence of specific antiviral therapy, vaccination is regarded as the most effective and reliable TBE prophylaxis. Although licensed inactivated vaccines are considered more effective [4], they have notable limitations. These include a complex immunization schedule requiring multiple primary doses and regular boosters every 3–5 years, and incomplete cross-protection against heterologous viral subtypes [5]. Consequently, despite vaccine availability, 12,000–15,000 TBE cases are registered annually [6,7], a burden partly attributable to suboptimal vaccination coverage in endemic areas. A part of these cases occurs in vaccinated individuals, who can develop severe, sometimes fatal or chronic, forms of TBE [8,9,10,11,12,13,14]. Furthermore, the incidence of TBE in endemic regions is increasing, and its geographical range is expanding [15,16,17,18,19]. In Russia, where the bulk of the virus distribution area is located, a majority of regions are recognized as endemic to TBE. As of 2024, 11 federal subjects of the Russian Federation were classified as highly endemic, with the Far-Eastern Federal District being at particular risk due to a rising incidence trend [20].

The currently licensed inactivated TBE vaccines are derived from formalin-inactivated strains of European (Neudöerfl) or Far-Eastern (Sofjin и 205) subtypes. They achieve 85–95% efficacy against homologous strains, but only 60–70% efficacy against Siberian subtype strains [21,22]. Thus, traditional vaccine platforms fail to adequately cover the full spectrum of natural viral genetic diversity. In contrast, mRNA technology has great prospects and allows precise design of the antigenic composition of the vaccine. Our previous data from an influenza virus model further demonstrate that the delivery of the target antigen using an mRNA platform induced a broader immune response with a significantly enhanced ability of antibodies to bind conserved hemagglutinin epitopes [23,24].

In recent years, mRNA vaccines have emerged as a revolutionary technology for infectious disease prophylaxis, demonstrating high efficacy, safety, and rapid development potential. This was exemplified by the successful deployment of COVID-19 vaccines (BNT162b2/Pfizer–BioNTech and mRNA-1273/Moderna) [25,26,27,28].

The development of mRNA vaccines against TBE offers new opportunities for disease prevention: such vaccines could offer higher immunogenicity, enable rapid adaptation to circulating viral variants, and be manufactured without the need for a live virus. Thus, an mRNA-based TBE vaccine represents a promising candidate to complement or potentially replace currently available vaccines. Furthermore, advances in mRNA vaccine development against other flaviviruses (e.g., Zika virus, dengue fever, yellow fever) [29] suggest the feasibility of this approach for TBE.

The TBEV genome encodes three structural proteins—the envelope (E), premembrane (prM), and capsid (C)—and seven non-structural proteins (NS1, NS2A, NS2B, NS3, NS4A, NS4B, and NS5). The E protein is the primary target for neutralizing antibodies; consequently, the detection of E-specific IgM and IgG in serum is used for both diagnosis and assessment of virus-specific immunity. In turn, the prM protein is essential as it forms a complex with the E protein to ensure proper maturation and presentation of the E protein on the virion surface, which is critical for a robust and effective immune response. Thus, in the current study we focused on determining the optimal antigen composition for the mRNA vaccine based on prM/E genes of various TBEV strains, which will provide wide cross-immunity against various TBEV subtypes. Furthermore, we evaluated the immunogenicity and protective efficiency of selected mRNA vaccine candidates.

## 2. Materials and Methods

### 2.1. Synthesis of mRNA Vaccine Candidates with PrM/E Antigen of Different TBEV Subtypes

The mRNA-LNPs encoding TBEV prM/E genes were synthesized in vitro, purified, and encapsulated into lipid nanoparticles via microfluidic mixing. For mRNA production, linearized plasmid constructions based on the pBAC vector (Lucigen) were used to obtain mRNA containing the genes of the PrM/E TBEV. Plasmids preparations were performed as previously described [23,30,31], utilizing open reading frames of the target antigens (Table 1). The mRNA encapsulation efficiency into lipid particles and concentration were determined by SYBR Green dye (SYBR Green I, Lumiprobe, Moscow, Russia), followed by monitoring the change in fluorescence.

### 2.2. Cell Lines

The following cell lines were used in this study: SPEV (BioLot, St Petersburg, Russia) for TBEV cultivation and SH-SY5Y (ATCC CRL-2266) for virus titration and assessment of virus-neutralization activity. Cells were cultivated at 37 °C and 5% CO_2_ in DMEM culture medium (PanEco, Moscow, Russia) with 10% fetal bovine serum (FBS) (HyClone, Logan, UT, USA).

### 2.3. Viruses

A total of 10 virus strains were used: 3 of Far-Eastern subtype (TBEV-FE, Sofjin, Krasny-Yar-8 and 205 strains); 1 of European subtype (TBEV-Eu, Zmeinogorsk-1 strain); 4 of Siberian subtype (TBEV-Sib, Lesopark-11, Aina-1448, Vologda-2 and Buzuuchuk (Kyrgyz line) strains); 1 of Baikal subtype (TBEV-Baik) 258-83 and 1 of 4th subtype (prototype strain 178-79). Virus titration was carried out using the plaque assay on SPEV cell monolayers using an overlay of 1.5% carboxymethylcellulose (CMC). After 96 h of incubation, the number of plaques was counted and calculated with the titers in plaque-forming units per milliliter (PFU/mL) using a standard formula accounting for the dilution of the virus suspension. Moreover, for the determination of virus titers in TCID50 titrations were performed on SH-SY5Y cell line.

### 2.4. Virus-Neutralization Assay

Animal sera were heat-inactivated at 56 °C for 30 min. Serial two-fold dilutions of the sera (starting from 1:10) were incubated with 100 TCID_50_ of corresponding TBEV strain at 37 °C for 1 h. Subsequently, the mixture was transferred onto a monolayer of SH-SY5Y cells in 96-well plates. The plates were incubated for 96 h at 37 °C with 5% CO_2_.

Each experiment included a virus control (without serum) and a cell control (without virus). The 50% neutralization titer (NT_50_) was determined using an MTT assay. An MTT solution (3 mg/mL) was added to the wells, and the plates were incubated for 3 to 5 h at 37 °C. After incubation, the medium was removed from the wells and replaced with DMSO. The optical density (OD) was measured using a plate reader at 570 nm.

### 2.5. Animal Models

All procedures with animals were carried out according to current guidelines on the care and use of laboratory animals and approved by the Local Ethics Committee of N.F. Gamaleya National Research Center for Epidemiology and Microbiology (protocol No. 76 from 24 September 2024). Female mice from line BALB/c weighing 18–20 g (aged 6–8 weeks) were used in the study. Mice were acquired from Cyagen (China) animal nursery. Animals were kept in the vivarium at 22 ± 2 °C, relative humidity of 50 ± 10% and 12/12 h light/dark cycle. All mice were subjected to a 7-day quarantine prior to the experiment. All manipulations were conducted under biosafety level 3 (BSL-3) laboratory conditions in accordance with the 3R principles (Replacement, Reduction, Refinement). If an animal lost more than 20% of its initial body mass or demonstrated severe neurological impairments, it was humanely euthanized by cervical dislocation under deep anesthesia. Animals would be anesthetized by intramuscular injection of zoletil–xylazine (45 mg/kg and 7.5 mg/kg, respectively).

### 2.6. LD_50_ Evaluation

To determine the LD_50_ five virus dilutions (10^5^, 10^3^, 10^1^, 5 and 2 PFU/animal) were prepared in sterile phosphate-buffered saline (PBS). Seven virus strains were used: Sofjin, Krasny Yar-8, 205, Zmeinogorsk-1, Lesopark-11, 178-79 and 258-83. Each virus dilution was administered intraperitoneally (i.p.) to a group of 6 mice in a volume of 100 μL. The control group (*n* = 6) received an equivalent volume of PBS. The general condition, behavior, and clinical symptoms of the animals were monitored for 12 days. The first death occurred on day 7 post infection. Body weight was measured once every 7 days until the first deaths occurred, and then daily thereafter.

### 2.7. Immunization by mRNA Samples

To evaluate the cross-reactive immune response, 6–8-week-old female BALB/c mice were immunized with one of ten different mRNA-LNP formulations (10 groups, *n* = 6) (Table 1). Each animal received two 50 μL intramuscularly injections containing 5 µg of mRNA, administered 14 days apart. The mRNA dose was selected based on our previous study [30]. Control mice received an equivalent volume of PBS via the same route. Two weeks after the booster immunization, blood samples were collected via cardiac puncture into clot activator tubes (VACUETTE^®^, Greiner Bio-One, Vienna, Austria) for serum isolation. The obtained serum was used to assess virus-neutralizing activity.

### 2.8. Protective Efficacy of mRNA Samples on Animal Models In Vivo

To evaluate the protective efficacy, female BALB/c mice aged 6–8 weeks were immunized twice with a 14-day interval. Five mRNA-PrM/E-LNP vaccine variants were used: Sofjin_FE, Krasny Yar_FE, 205_FE, 178-79_4 and a mixture of Sofjin_FE + Krasny Yar_FE; for each vaccine variant, a group of 52 mice was immunized (5 groups, *n* = 52). Before the challenge infection, the immunized mice from each vaccine group were redistributed. Therefore, 52 immunized mice were divided into eight subgroups (*n* = 6): seven groups for infection with different TBEV strains (one group = one virus) and one control group of immunization. Additionally, four mice were sacrificed prior to infection to collect serum for analysis. In parallel, control groups (injected with PBS) were formed for their consequent infection with seven TBEV strains: Sofjin_FE, Krasny Yar-8_FE, 205_FE, Zmeinogorsk-1_Eu, Lesopark-11_Sib, 178-79_4, 258-83_Baik (7 groups, *n* = 6). In 14 days after the second immunization, the animals were infected with TBEV at doses of 100 LD_50_. LD_50_ was calculated by the Kerber method based on the previously determined titer of each strain. After infection, animals were weighed daily and their clinical condition was evaluated, documenting the following parameters: body mass, motor activity, presence of neurological symptoms (pareses, paralyses, seizure activity) and survival rate. The animals were monitored for 21 days.

### 2.9. Estimation of Viral Load in Animal Organs by Quantitative PCR

Viral load and virus replication were assessed using quantitative reverse transcription PCR (RT-qPCR) with hybridization-fluorescence detection.

Organ samples (brain, heart, liver, spleen) were homogenized in 1 mL of PBS with protease inhibitors (cOmplete ULTRA Tablets—Mini, EDTA-free, EASYpacks) using aTissueLyser II (50 Hz, 5 min). The homogenates were centrifuged for 20 min at 12,000× *g* and at 4 °C. The supernatants were collected and stored at −80 °C.

Total RNA was extracted from 100 μL of tissue lysate using ExtractRNA reagent (Eurogen, Moscow, Russia) according to the manufacturer’s guide. Reverse transcription and qPCR were conducted using a commercial kit for TBEV RNA detection AmpliSense^®^ TBEV, *B. burgdorferi sl*, *A. phagocytophillum*, *E. chaffeensis*/*E. muris-FL* (Central Research Institute of Epidemiology of Rospotrebnadzor, Moscow, Russia) according to the manufacturer’s guide. The amplification conditions were as follows: 50 °C for 15 min and 95 °C for 5 min, followed 5 cycles of 95 °C—10 s, 60 °C—35 s and 72 °C—15 s, followed 45 cycles 95 °C—10 s, 56 °C—35 s and 72 °C—30 s.

Data analysis was performed with the CFX 96 Real-Time PCR Detection System software (CFX Manager 3.1, Bio-Rad, Hercules, CA, USA), monitoring the accumulation of the FAM fluorescent signal, which corresponds to the accumulation of TBEV cDNA.

### 2.10. ELISA

Indirect enzyme-linked immunosorbent assay (ELISA) was used to evaluate the titers of specific anti-TBEV antibodies in serum of immunized mice. Recombinant E proteins were used as the antigen. E proteins obtained from various TBEV strains were used as the antigen: 205_FE), Lesopark-11_Sib, Vologda-2_Sib and 886/84_Baik. A total of 0.5 μg of antigen in purified water was added into the wells of a 96-well immunoassay plate (NEST, Beijing, China). The plate was incubated at 4 °C for 16 h, followed by quintuple washing with PBST (0.01 M PBS with 0.05% Tween-20). Nonspecific binding was blocked with 1% solution of bovine serum albumin (BSA) in PBS (BSA-PBS) at 37 °C for 1 h, followed by subsequent washing with PBST. Consequently, 0.1 mL of investigated serum samples were added in different dilutions in 1% BSA-PBS and incubated at 37 °C for 1 h, once again followed by subsequent washing with PBST. After that, 0.1 mL of HRP-conjugated anti-mouse IgG in BSA-PBS were added and incubated at 25 °C for 45 min. Finally, after another washing with PBST, 0.1 mL of TMB were added and incubated for 10 min at room temperature, then stopped with 0.1 mL of 1 M sulfuric acid.

Endpoint antibody titers (EAT) were determined in serially diluted serum samples. Intact mouse serum (PBS group) was used as a negative control (K-) at dilutions ranging from 1:100 to 1:100,000 with a 10-fold step in PBST–BSA.

Results were interpreted based on the signal-to-noise ratio (S/N), where S represents the optical density (OD) value of the sample and N is the mean OD value of the negative control. Samples with value of S/N > 3 were considered positive.

### 2.11. Statistical Analysis

Statistical analysis, as well as plotting heat maps and graphs, was performed using GraphPad Prism 6.0. Virus-neutralizing antibody levels were compared using the Kruskal–Wallis test and Dunn’s multiple comparison post hoc test. Data are expressed as the mean ± standard error of the mean (SEM) from at least three independent experiments. Statistical significance was set at *p* < 0.05. ELISA data are presented as geometric means from at least three independent experiments.

## 3. Results

### 3.1. Preparation of Linear Plasmid Constructions for Synthesizing mRNA Vaccine Candidates

We designed and synthesized a panel of ten mRNA-LNP constructs encoding the PrM/E proteins of distinct TBEV strains. The panel was designed to encompass the primary genetic diversity of the virus, including next representatives: three related to the Far-Eastern subtype (Sofjin, Krasny Yar-8, 205), one to the European subtype (Zmeinogorsk-1), four to the Siberian subtype (Lesopark-11, Aina-1448, Vologda-2, Buzuuchuk) and one each to the fourth subtypes (178-79) and Baikal subtype (258-83). For the selection of TBEV strains for vaccine mRNA candidate development, we relied on virus-neutralization data published previously [32], as well as the spectrum of currently dominant subtypes. Thus, according to earlier experiments, immunization with the Krasny Yar variant of TBEV, which belongs to subtype 1, provided the highest virus-neutralizing activity. The main variants used in Russian vaccines are strains belonging to subtype 1 (Sofjin and 205), while, among currently circulating strains, representatives of the third subtype are increasingly gaining dominance [15,32].

To obtain the mRNA-PrM/E-TBEV-LNP in this work, linear plasmid constructions pBAC were used as described previously [30]. These constructions contained open reading frames for the investigated TBEV antigens. To ensure stability of the poly(A) tract during vector propagation in bacterial culture, the tract was divided into segments. The first 30 adenines were divided with the next 70 by a 10-nucleotide spacer. All plasmid constructions were verified by Sanger sequencing.

### 3.2. Screening of mRNA Vaccine Candidates

**Figure 1:**
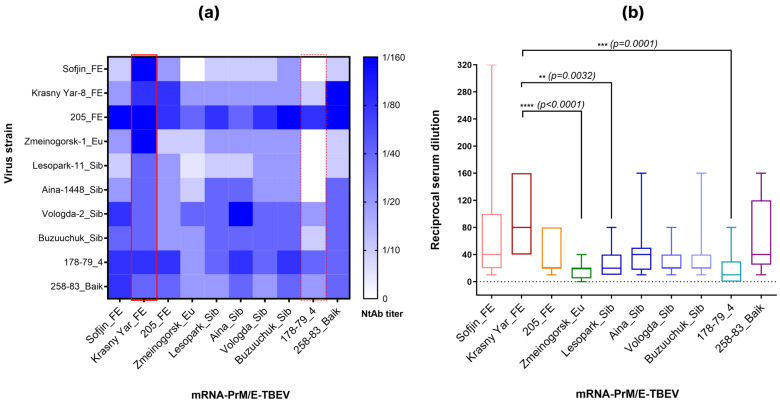
Evaluation of cross-virus-neutralizing activity of mice sera. (**a**) Heat maps of virus-neutralization titer. The solid red line indicates the leading vaccine candidate. The dashed red line indicates the variant with the least favorable characteristics. (**b**) comparison of virus-neutralization titers in sera of mice immunized by mRNA-PrM/E-TBEV. *p*-values were calculated using the Kruskal–Wallis test (** *p* < 0.01, *** *p* < 0.001, **** *p* < 0.0001).

Immunization with the ten mRNA-PrM/E-TBEV candidates elicited formation neutralizing antibodies in mice. Sera from immunized animals neutralized both homologous and heterologous TBEV strains. Average 50% neutralizing serum titer (NT_50_) ranged from 1:16.9 to 1:84.4, signifying presence of neutralizing antibodies (Figure 1b, Table 2). The highest efficacy in regard to cross-neutralizing activity was exhibited by mRNA-PrM/E-Krasny Yar-8_FE, while mRNA-PrM/E-Zmeinogorsk_Eu and mRNA-PrM/E-178-79_4 have shown the lowest efficacy (Figure 1, Table 2). A notable observation was that for several candidates, neutralization titers against heterologous viral subtypes were higher than those against the homologous subtype used for immunogen design. This pattern aligns with prior clinical and experimental observations in TBEV immunology, where immunization with one subtype (e.g., European) can induce a serum response with superior activity against a heterologous subtype (e.g., Far-Eastern) [4].

Based on this data, the following mRNA-PrM/E-TBEV candidates were selected for further evaluation of protective efficacy: Sofjin_FE, Krasny Yar-8_FE and 205_FE (all belonging to the Far-Eastern subtype), as well as mRNA-PrM/E-TBEV-178-79_4. It is, therefore, anticipated that it will be confirmed that the vaccine mRNA variants based on TBEV strains of subtype 1 induce the highest immune response compared to the mRNA variant based on strain subtype 4.

### 3.3. Evaluation of Protective Efficacy of the mRNA Vaccine

**Figure 2:**
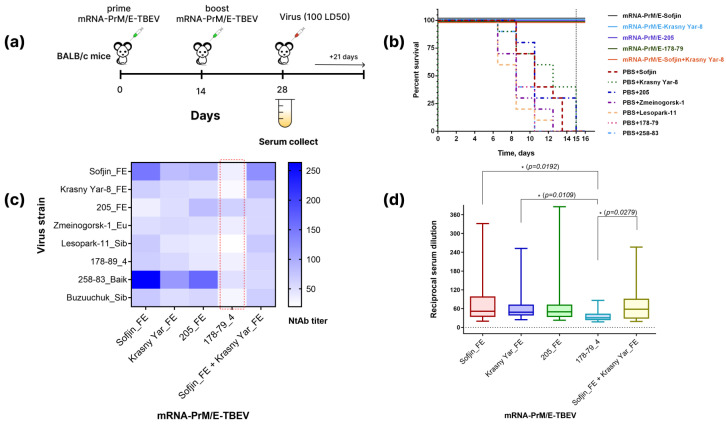

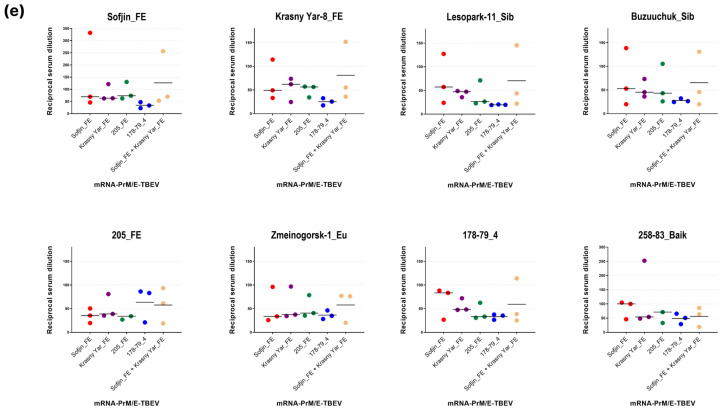
Evaluation of the protective efficacy of mRNA-PrM/E-TBEV-LNP. (**a**) Scheme of experiment; (**b**) survival rates of animals in groups vaccinated with the mRNA vaccine and in control groups (PBS); (**c**–**e**) results of virus-neutralizing activity of sera from immunized animals. The dashed red line indicates the variant with the least favorable characteristics. Shown *p*-values were calculated using the Kruskal–Wallis test (* *p* < 0.05). Survival analysis was performed using the Kaplan–Meier method for immunized mice infected with seven TBEV strains at a dose of 100 LD_50_.

To evaluate protective efficacy, groups of BALB/c mice were immunized by five different mRNA-PrM/E-LNP vaccines encoding the prM/E proteins of the following strains: Sofjin_FE, Krasny Yar-8_FE, 205_FE, 178-79_4, or a combination of Sofjin_FE and Krasny Yar-8_FE. Animals were immunized with mRNA (5 μg per animal) twice with a 14-day interval, while control animals received PBS. Two weeks after the booster immunization and prior to challenge, four mice from the vaccine group were euthanized for serum collection to assess anti-TBEV antibody titers and virus-neutralizing activity (VNA). The remaining mice were then challenged intraperitoneally with one of seven TBEV strains (Sofjin_FE, Krasny Yar-8_FE, 205_FE, Zmeinogorsk-1_Eu, Lesopark-11_Sib, 178-79_4 and 258-83_Baik) at a dose of 100LD_50_. The experimental design is summarized in Figure 2a.

During the observation period, animal deaths were recorded only in the control groups (PBS). The onset of symptoms occurred between days 7 and 10; mortality was observed by day 15 post infection in all control groups. The survival rate of animals in the mRNA groups was 100% (Figure 2b).

VNA titer of sera of immunize mice was evaluated against eight different TBEV strains (Sofjin_FE, Krasny Yar-8_FE, 205_FE, Zmeinogorsk-1_Eu, Lesopark-11_Sib, 178-79_4, 258-83_Baik, Buzuuchuk_Sib); the results are shown in Figure 2c–e. As in the first experiment, analysis of serum samples from immunized animals has shown cross-VNA against eight TBEV strains (Figure 2c).

**Figure 3:**
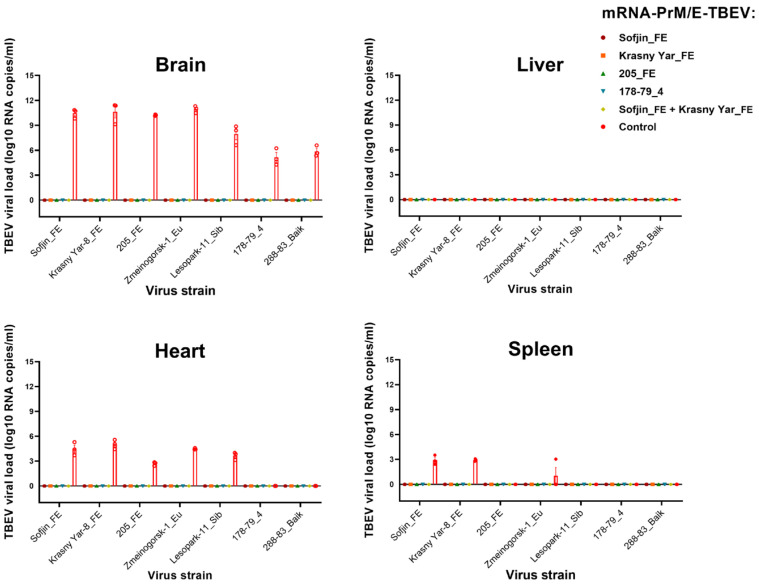
Levels of TBEV viral loads across specimen types.

The viral load data, as determined by one-step RT-qPCR, correlated with the results of survival. Viral RNA was detected in homogenates of the brain, heart, and spleen of mice from the control (PBS) group, while viral RNA was not detected in any organs from mice immunized with the mRNA vaccine (Figure 3). As anticipated, the highest viral RNA load was observed in brain homogenates; however, no virus was detected in the liver samples.

Antibody levels in the serum of immunized mice were assessed by an in-house ELISA. Four different E proteins of TBEV were used in analysis—205_FE, Lesopark-11_Sib, Vologda-2_Sib, 886/84_Baik—to assess specific antibodies against these variants.

**Figure 4:**
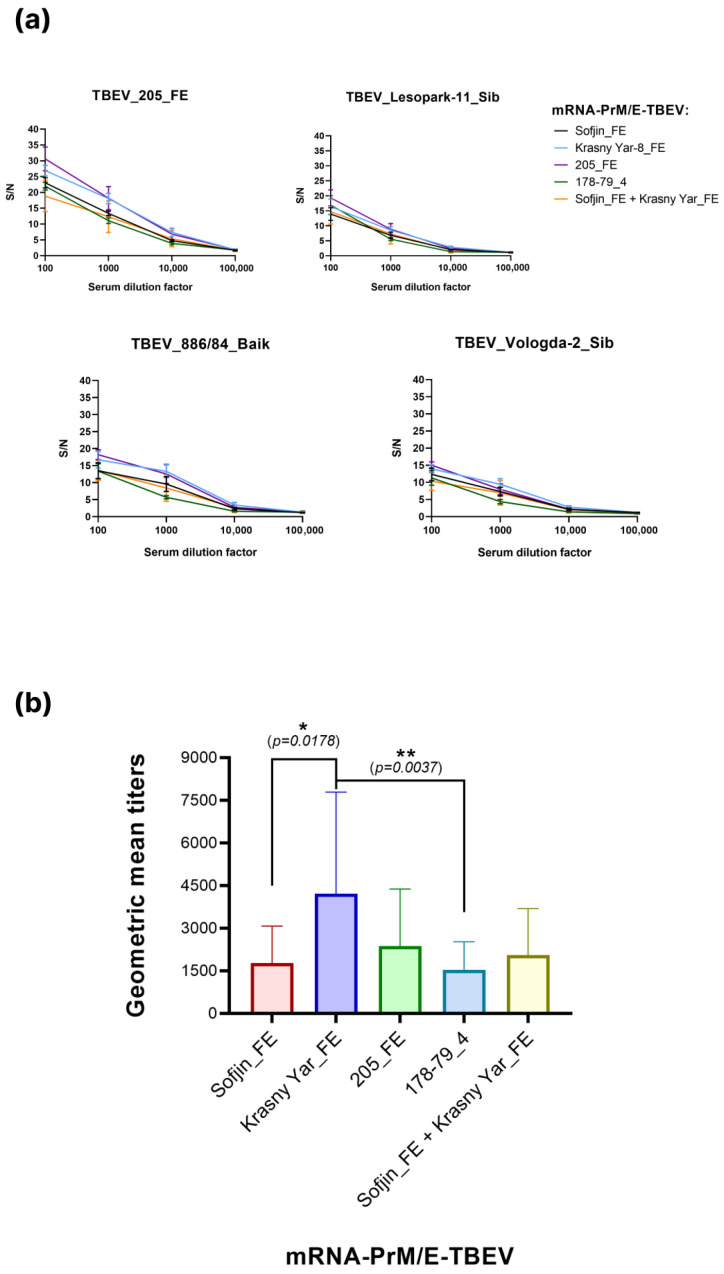
Evaluation of immunogenicity of mRNA-PrM/E-TBEV by ELISA. (**a**) S/N data presented as mean ± standard error of mean; (**b**) average geometric mean of antibody titers. Shown *p*-values were calculated using the Kruskal–Wallis test (* *p* < 0.05, ** *p* < 0.01).

All serum samples tested positive (S/N > 3) against all four antigens (Figure 4a). The strongest immune response was observed when the E protein from the 205_FE strain was used as the antigen.

To determine the antibody titer, the geometric mean titer (GMT) was calculated from all serum dilutions that yielded a positive result (Figure 4b). The endpoint titers varied depending on the antigen used.

In summary, immunization with the mRNA candidates elicited specific antibodies against all target antigens.

The immunogenicity of the mRNA vaccine candidates was assessed by measuring antigen-specific antibody levels in mouse sera using ELISA. The candidate based on the Krasny Yar-8_FE TBEV strain demonstrates the highest antibody titers against a panel of heterologous antigens (Table 3), indicating its potential to elicit a broad cross-reactive immune response.

Comprehensive in vitro analysis of sera from immunized mice demonstrated the presence of cross-neutralizing activity against both homologous and heterologous TBEV subtypes. In the assessment of binding antibodies, cross-reactivity with all four antigen variants was also observed. Collectively, these data identify the mRNA-PrM/E-Krasny Yar_FE construct as the lead candidate, demonstrating superior performance across all evaluated parameters.

## 4. Discussion

Currently, no specific antiviral therapy is available for arboviral flavivirus infections. Thus, vaccination remains the primary strategy for prophylaxis and control of infectious diseases. As of date of publication, FDA has only approved either attenuated (for YFV) or inactivated (JEV and TBEV) vaccines. Inactivated vaccines are generally considered safer than attenuated ones due to the former’s inability to cause infection. However, if the inactivation procedure is not conducted thoroughly during manufacturing of the vaccine, there is a possibility of introducing infection infectivity viral particles into the vaccinated person’s body [33,34]. On the contrary, if the inactivation protocol is too harsh, it may lead to an inverse effect, damaging the virus-neutralizing epitopes and leading to insufficient nAb generation.

Presently, eight vaccines for TBE prophylaxis in children and adults are certified in the Russian Federation [35]. All of them are based on Far-Eastern or European strains and involve formalin inactivation of the virus.

Literature data indicate that the cross-immunogenicity and protective efficacy conferred by different TBEV strains in vaccines against heterologous strains vary significantly and can be incomplete even among viruses of the same subtype [1,36]. This shows the necessity of further studies which will allow selecting antigens with the widest possible scope of cross-immune response. This study presents a comprehensive characterization of a novel mRNA vaccine candidate against TBEV. The vaccine candidates demonstrate high immunogenicity and efficacy in animal models. The primary aspect of this work is the comprehensive approach to the determination of the vaccine’s antigen composition, taking all described subtypes of TBEV into account. Also, the choice of TBEV strains for synthesis of mRNA samples was based on earlier virus-neutralization data [32]. Consequently, we generated and evaluated a panel of ten mRNA-PrM/E-TBEV constructs representing five TBEV subtypes. Through this approach, it is possible to include the maximum diversity of circulating virus strains, which is especially important considering high (up to 16%) variability of amino acid composition in E proteins between subtypes [37,38,39,40,41].

The data in the conducted research demonstrate protective efficacy of all candidate mRNA-PrM/E-TBEV variants, providing 100% survival rate when infecting with both homologous and heterologous TBEV variants. However, in vitro analysis of sera of immunized mice signifies considerable differences in VNA and antibody titers.

Regarding the vaccine’s immunogenicity, it should be noted that mRNA-PrM/E—Krasny Yar-8_FE demonstrates high immunogenicity, significantly different from other candidates. In vaccine development, particularly mRNA-based ones, determination of the strain which can provide the highest efficacy and widest scope of defense is one of the key steps.

Even though Krasny Yar-8 is attributed to the Far-Eastern TBEV subtype—same as Sofjin and 205 conventionally used in inactivated vaccines—it was first isolated in the Irkutsk District where the Siberian subtype is regarded as endemic. It is theorized that due to numerous consequent recombinations between circulating strains of various subtypes [42], this variant procured a certain combination of properties allowing for induction of neutralizing antibodies with a higher scope of defense. Thus, application of such antigens in the vaccine may increase its efficacy against contemporary virus variants.

Information about any development of an mRNA vaccine against TBEV remains sparse. Still, successful implementation of this technology against other flaviviruses shows that such a development is promising. Successful examples include the mRNA Zika virus vaccine developed by Moderna showing efficacy in preclinical trials [43], as well as a tetravalent vaccine against the Dengue virus facilitating immune response against all four virus serotypes [44].

The results of this study allow for better understanding of the mRNA platform’s capabilities in prophylaxis of flavivirus infections. Further research should be aimed at optimizing the vaccine’s construction, investigating sustained immunity and assessment of its efficiency against contemporary circulating TBEV strains.

## 5. Conclusions

The novel mRNA vaccine candidate described here holds significant promise for TBE prevention in regions with co-circulating virus subtypes. Furthermore, it may provide a platform for developing universal vaccines against flaviviral infections.

## 6. Patents

A patent application resulting from the work reported in this manuscript has been filed (Application No. 2025136593, filed on 17 December 2025). 

## Figures and Tables

**Table 1:** Constructions of mRNA-PrM/E-TBEV-LNP created on the basis of TBEV strains of various subtypes and used in the current work.

TBEV Strain	TBEV Subtype, Place and Source of Strain Isolation	GenBank
Sofjin	1 (TBEV-FE) Far East, Russia, a patient’s blood sample	KC806252.1
Krasny Yar-8	1 (TBEV-FE) Irkutsk region, Russia, tick *I. persulcatus*	PX467450.1
205	1 (TBEV-FE) Khabarovsk region, Russia, tick *I. persulcatus*	JX498939.1
Zmeinogorsk-1	2 (TBEV-Eu) Altai region, Russia, tick *I. persulcatus*	KY069124.1
Lesopark-11	3 (TBEV-Sib) Novosibirsk region, Russia, tick *I. persulcatus*	KJ701416.1
Aina/1448/A-53	3 (TBEV-Sib) Irkutsk region, Russia, a patient’s blood sample	JN003206.1
Vologda-2	3 (TBEV-Sib) Vologda Oblast, Russia, tick *I. persulcatus*	AF229364.1
Buzuuchuk	3 (TBEV-Sib) Kyrgyzstan, tick *I. persulcatus*	KJ626343.1
178-79	4 (the original subtype) Irkutsk region, Russia, tick *I. persulcatus*	EF469661.1
258-83	5 (TBEV-Baik) Republic of Buryatia, Russia, tick *I. persulcatus*	MW524087.1

**Table 2:** Titer of virus-neutralizing antibodies (NT_50_) in serum of mice immunized with mRNA-PrM/E-TBEV.

mRNA-PrM/E-TBEV-LNP	Average NT_50_	Median [Q1, Q3 ^1^]
mRNA-Sofjin_FE	75.6	40 [20, 100]
mRNA-Krasny Yar_FE	84.4	80 [40, 160]
mRNA-205_FE	43.9	20 [20, 80]
mRNA-Zmeinogorsk_Eu	16.9	20 [5, 20]
mRNA-Lesopark_Sib	28.3	20 [10, 40]
mRNA-Aina_Sib	46.0	40 [17.5, 50]
mRNA-Vologda_Sib	30.8	20 [20, 40]
mRNA-Buzuchuuk_Sib	38.3	20 [20, 40]
mRNA-178-79_4	22.3	10 [0, 30]
mRNA-258-83_Baik	63.9	40 [25, 120]

^1^ Q1—25th percentiles; Q3 —75th percentiles.

**Table 3:** Serum IgG antibody titers against TBEV E protein in mRNA-vaccinated mice.

mRNA-PrM/E-TBEV-LNP	Mean	Geometric Mean	Lower 95% CI GMT ^1^
mRNA-Sofjin_FE	3250	1778	1027
mRNA-Krasny Yar_FE	6625	4217	2283
mRNA-205_FE	4375	2371	1284
mRNA-178-79_4	2688	1540	939.1
mRNA-Sofjin_FE + Krasny Yar_FE	3813	2054	1141

^1^ CI—confidence interval; GMT—geometric mean.

## Data Availability

The data presented in this study are available on request from the corresponding author.
